# Influence of MAD Application on Episodes of Obstructive Apnea and Bruxism during Sleep—A Prospective Study

**DOI:** 10.3390/jcm11195809

**Published:** 2022-09-30

**Authors:** Monika Wojda, Jolanta Kostrzewa-Janicka

**Affiliations:** Department of Prosthodontics, Medical University of Warsaw, 02-097 Warsaw, Poland or

**Keywords:** obstructive sleep apnea, sleep bruxism, mandibular advancement devices

## Abstract

The condition of sleep bruxism (SB) is defined by many authors as the body’s response to obstructive sleep apnea (OSA). In the conservative treatment of OSA, mandibular advancement devices (MADs) have found their application. The aim of the study iso assess the impact of MADs on the occurrence of episodes and the intensity of OSA and SB. The study sample consisted of eight patients with OSA and SB diagnosed with these conditions on the basis of clinical examinations and polysomnography (PSG). The prospective study was designed to assess the use of MADs for OSA and SB. MADs were prepared for the patients who subsequently underwent control examinations after one week of wear, and another PSG (PSG II) with an MAD was performed in conditions resembling the first qualification examination (PSG I). The same parameters were assessed in both PSG examinations. Following treatment with the MAD, a favorable lowering of the mean values of the examined parameters was observed. The statistically significant differences were demonstrated only for the apnea–hypopnea index (AHI), the oxygen desaturation index (ODI), and the number of apneas and hypopneas, obstructive apneas, apneas in OSA, and phasic episodes of bruxism. The application of MADs in patients with OSA has a beneficial effect on the same manifestations of OSA and SB, even though only the number of phasic episodes of bruxism was statistically significant.

## 1. Introduction

Obstructive sleep apnea (OSA) occurs in about 9–38% of the adult population [1], and sleep bruxism (SB) occurs in about 13%. Both of these medical conditions occur during sleep, but associations between them have never been definitely explained [2,3]. OSA is characterized by episodes of complete collapse (apnea) or narrowing (hypopnea) of the upper respiratory tract at the pharyngeal level whilst maintaining and, in most cases, even increasing the functioning of the respiratory muscles. Such episodes generally lead to diminished arterial blood oxygen saturation and usually cause the patient to wake up, although they are often unaware of that fact despite loud snoring, as the respiration is being restored [4]. According to the 2018 International Consensus, bruxism is defined as masticatory muscle activity that occurs during sleep (characterized as rhythmic or non-rhythmic) and wakefulness (characterized by repetitive or sustained tooth contact and/or by bracing or thrusting of the mandible), respectively [2]. 

The gold standard in the diagnostics of OSA and SB is polysomnography (PSG), which enables the observation of both conditions simultaneously. Additionally, audio–video recordings facilitate the verification of the character of sounds that can be heard during the examination (gnashing, tapping, snoring, and others) and the kinds of movements (sighing, swallowing, coughing, myoclonus, and others). 

In the conservative treatment of OSA, devices that produce continuous positive airway pressure (CPAP) are the method of choice. In individuals with a mild and moderate type of OSA and in patients with severe OSA who fail to tolerate treatment with CPAP, mandibular advancement devices (MADs) have also found their application [5,6,7,8]. MADs work on the principle of maintaining the mandible in the protruded position, displacing the tongue forward by means of the genioglossus muscle and changing the position of the hyoid bone to widen the upper respiratory tract [6]. Lavigne et al. presented a sequence of physiological events in OSA. Before the masticatory muscles become active as a consequence of the activity of the sympathetic system, the pulse becomes more rapid and a rise in alpha activity is observed on an EEG, after which the suprahyoid muscles that lower the mandible contract, which increases the airway patency. Then, the masseters contract to produce the phenomenon of grinding. For many authors, bruxism, as masticatory muscle activity, is the body’s response to the increase in sympathetic activity [9,10,11,12]. Thus, investigating whether MAD-wearing individuals still experience episodes of bruxism merits research. In view of the inconclusive results of many studies on the association of bruxism with sleep apnea, the impact of devices used in the treatment of OSA on both of these conditions should be examined [9,11,13]. The aim of the study was to evaluate the effect of MADs on the occurrence of episodes and the severity of OSA and SB.

## 2. Materials and Methods

The study protocol gained the approval of the Bioethics Committee of the Medical University of Warsaw (KB/139/2018). A group of 8 patients (all male) with OSA and SB, who had been diagnosed on the basis of clinical and polysomnographic examinations, constituted the study material. At the Sleep Disorder Clinic of the Internal Diseases, Pneumology, and Allergology Department, the patients completed their own questionnaires concerning obstructive sleep apnea, which additionally included the Epworth Sleepiness Scale (ESS) and bruxism prevalence questionnaires. The ESS was based on the patient’s self-assessment to determine the probability of falling asleep in eight different life situations. The respondent had four options to choose from, with 0 indicating an inability to fall asleep in a given situation and 3 indicating a high probability of falling asleep. The number of points to score was between 0 and 24. Excessive daytime sleepiness, which is one of the criteria for suspected OSA, was determined with scores higher than 10. The bruxism questionnaire consisted of five questions concerning the occurrence of clenching and grinding in patients in a waking state and during sleep, as well as a reported sensation of stiffness or clenched jaws upon waking in the morning. Nocturnal grinding of the teeth during sleep was diagnosed either on the basis of the patients’ own convictions that they indeed suffered from this condition or because somebody had told them about it [14].

The patients with suspected OSA and SB who qualified for the study (had positive questionnaire results for OSA and SB) underwent the first PSG examination with electrodes additionally fixed on the masseters to assess SB (PSG I). The occurrence of the episodes of bruxism was verified with the analysis of audio–video recordings to distinguish the types of audible sounds (gnashing, tapping, snoring, etc.) and to eliminate certain types of movements not related to SB (sighing, swallowing, coughing, myoclonus, and others). The PSG documentation included a six-channel electroencephalogram, a bilateral electrooculogram, an electromyogram from the chin, anterior tibial muscle, and masseter area bilaterally, an electrocardiogram, a record of chest and abdomen movements, position of the body during sleep, airflow through the airways, and audio–video recordings. The PSG examination and the audio–video recordings were assessed by two independent physicians, taking into account the PSG assessment criteria for OSA and SB. Both examiners had received the same training. The evaluation of the frequency of the episodes of bruxism as registered during the PSG was performed in accordance with the criteria set by Lavigne et al. [15]. For this purpose, the bruxism episode index (BEI), phasic, tonic, and mixed episodes of bruxism, and a summary number of all types of episodes of bruxism were considered. The episodes of apnea were assessed on the basis of the American Academy of Sleep Medicine (AASM) criteria [16]. To assess OSA in the PSG, the apnea–hypopnea index (AHI), the oxygen desaturation index (ODI), the number of all types of apnea–hypopnea episodes, the number of obstructive apnea, mixed apnea, hypopnea, and apnea episodes, and the mean and minimal values of SpO_2_ were calculated.

For patients diagnosed with OSA and SB on the PSG I, a dental examination was conducted to assess the number and condition of the teeth retained in the mouth, the periodontal condition, and the presence of temporomandibular dysfunctions so that an MAD could be provided. 

Following the analysis of inclusion and exclusion criteria, the study group consisted of eight patients with moderate-to-severe forms of OSA and sleep bruxism (Table 1), who were subsequently provided with MADs. 

**Figure 1:**
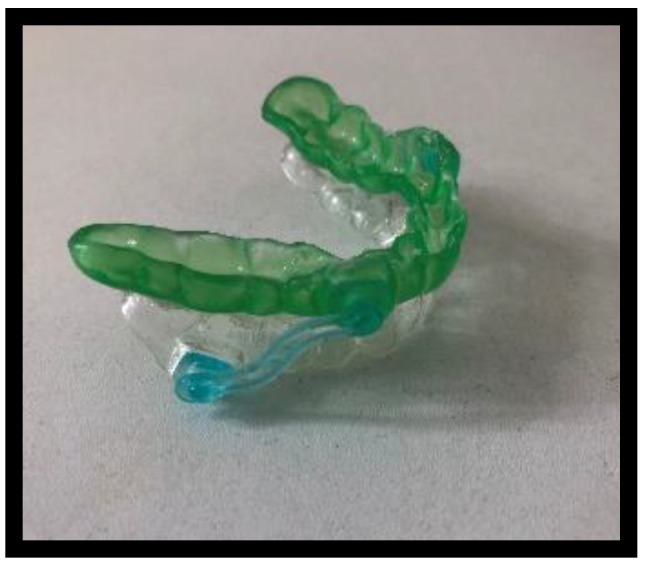
Mandibular advancement devices, Silensor-sl (Erkodent).

In this study, Silensor-sl (Erkodent) single-unit mandibular protruding devices were used, consisting of splints for the upper and lower dental arch with connectors in a registered constructive bite, enabling unobstructed mouth breathing. The constructive bite was determined with the George bite registration device (Scheu Dental Technology) with the advanced mandibular position at 60% of the maximum protrusion. The splint, performed with the pressing technique, covered the occlusal surfaces and incisal edges of all teeth, including the vestibular regions to ¾ of their height and the lingual surfaces of the teeth, extending to the alveolar mucosa of the right-sided ridge of the oral cavity (Figure 1). 

For patients who had been provided with their MADs, a checkup examination and a second PSG with the MAD in the mouth (PSG II) were arranged. The first checkup visit took place a week after the patients had been given their MADs. During that visit, the device was adjusted to relieve the areas of excessive pressure to improve the comfort of wear. The adaptation period lasted a month, after which the second PSG with an MAD in the mouth was performed. The conditions of this second examination were similar to those of the first qualifying examination (PSG I). During the PSG II, the same parameters were examined as in the previous PSG examination. 

The statistical analysis consisted of a comparison of the effects of the MAD application on the eight patients in the scope of fourteen parameters. The data included parameter values of the same patient prior to and following an MAD application obtained in the PSG I and PSG II examinations. The analysis was based on a Student’s *t*-test for the dependent groups or a Wilcoxon signed-rank test. All the examined variables were measured on a continuous scale. In the case of a normal distribution, the Student’s *t*-test for the dependent variables was employed as the test of choice. Otherwise, a non-parametric Wilcoxon test was used. The analysis started with checking the distribution, and then the proper test was employed. Additionally, Wilcoxon tests were used for all of the examined parameters to confirm or reveal other significant associations. The correlations of the dependent samples were analyzed by determining the Pearson correlation coefficient.

## 3. Results

The PSG results at the baseline and after the MAD application indicate positive changes in the OSA and SB parameters, as revealed during the PSG II in comparison with the baseline (PSG I) (Table 2). The MAD application resulted in a favorable decrease in the mean values of the AHI and ODI indices, the number of apnea and hypopnea episodes, the number of hypopneas, the number of apneas, obstructive apneas, and mixed apneas, the BEI, the number of bruxism episodes, and the number of phasic, tonic, and mixed episodes of bruxism, and a favorable increase in the mean values of the minimal and mean saturation.

The Statistical analysis with the Student’s *t*-test and the Wilcoxon test was sufficient to state that the statistically significant changes of the mean values of the examined parameters after the MAD application were revealed only in relation to the AHI and ODI, the number of apneas and hypopneas, the number of obstructive apneas, the number of apneas, and the number of episodes of phasic bruxism (Table 3). As for the MAD application, the following statistically significant reductions were noted: the AHI was reduced on average by 11.05 ± 8.35 (*p* = 0.007); the ODI was reduced on average by 15.28 ± 12.0 (*p* = 0.009); the number of apneas and hypopneas was reduced on average by 90.75 ± 59.07 (*p* = 0.003); the number of obstructive apneas was reduced on average by 72.86 ± 68.94 (*p* = 0.020); the number of apneas was reduced on average by 90.5 ± 74.09 (*p* = 0.011; the number of phasic episodes of bruxism was reduced on average by 6.38 ± 8.75 (*p* = 0.034).

For the number of mixed apneas, no statistically significant differences were found between the number of mixed apneas on the PSG with and without an MAD (PSG I and II) (*p* = 0.24). Similar results were obtained for the hypopneas (*p* = 0.78), the mean SpO_2_ (*p* = 0.230), the minimal SpO_2_ (*p* = 0.155), the BEI (*p* = 0.77), the number of episodes of bruxism (*p* = 0.150), the number of tonic bruxism episodes (*p* = 0.401), and the number of mixed bruxism episodes (*p* = 0.068). 

In order to verify whether extreme and outlier values impacted the significance of the results, the tests were repeated, each time following the elimination of one case. The elimination of extreme and outlier cases did not alter the results, meaning that no additional significant correlations were found.

A very strong correlation in the AHI with and without an MAD was revealed (r = 0.898, *p* = 0.002). Similarly, strong correlations relating to the examinations with and without the device were found for the following: the ODI (r = 0.740, *p* = 0.036), the number of apneas and hypopneas (r = 0.885, *p* = 0.003), and the mean SpO_2_ (r = 0.846, *p* = 0.008). Another finding was an almost functional association of the number of rhythmic episodes of bruxism with and without an MAD and hypopneas with and without an MAD at r = 0.948 (*p* = 0.000) and r = 0.905 (*p* = 0.002), respectively. All of these significant correlations were positive (Table 4). Yet, a much larger sample is required to examine the effect of the improved parameters on the therapeutic effectiveness of MADs. 

## 4. Discussion

There are studies confirming an association between OSA and SB [9,11,13,17,18] and the therapeutic efficacy of MADs in the management of OSA [19,20,21]. Hosoya et al. and the authors of the present study demonstrated a positive correlation between the incidence of OSA and the episodes of sleep bruxism in a group of patients diagnosed with OSA during a PSG examination. The present study revealed that sleep bruxism episodes occurred decisively more often in patients with OSA than in the controls, and they were observed during microarousals as a consequence of an OSA episode [13,17]. In their studies, Aarab et al. found a difference between the baseline AHI parameters and the results after treatment with continuous positive airway pressure (CPAP) above 20 and a difference of 15 in the number of apnea episodes before and after treatment with an MAD, which indicates that the number of apnea episodes decreased significantly in comparison with the baseline results for both the MAD and CPAP treatment modalities. The differences in the AHI were significant when comparisons were performed with a placebo splint group. Additionally, excessive daytime sleepiness manifested a downward tendency when both types of devices were used [19]. 

The association between OSA and SB, as well as the therapeutic efficacy of MADs in patients with OSA, prompted the commencement of studies on the impact of MADs on SB. Because of the varied etiology of bruxism, patients can benefit from the following three therapeutic strategies: occlusal, pharmacological, and behavioral psychotherapy. In occlusal therapy, we can use nightguards, which protect the patient’s dentition from wear due to attrition, relax the muscles, and reduce the effects of parafunctions. Since they do not protrude the mandible, it would seem that they should not impact on the occurrence of OSA and, as a consequence, SB, provided that in this case, apnea is due to bruxism. Management based on a single method is usually insufficient to protect the peripheral tissues due to SB. Studies have demonstrated the therapeutic efficacy of MADs with regard to SB patients with OSA [22,23,24,25,26,27]. There are also studies that confirm the superior effectiveness of MADs in reducing the number of SB episodes in comparison with the application of occlusal splints [25,26,27]. Martynowicz et al. reported a reduction in BEI in both groups of patients treated with CPAP and MADs [22]. It has also been demonstrated that a variety of intra-oral devices lower the frequency of bruxism episodes [23,24], although in the presented study, only the number of episodes of phasic bruxism was reduced. Several factors could have influenced the obtained results. One of them could be insufficient mandibular protrusion. Another was that there was no correlation between OSA and SB in the examined patients. Although both conditions were confirmed in the study group subjects, it is not known if SB was due to OSA; thus, the effect of the MADs on bruxism cannot be verified. The absence of positive effects of the MADs on SB may be due to an insufficient number of PSG examinations with the MADs in the mouth and patients’ individual determinants. 

A prerequisite for an MAD application in an OSA treatment is meeting specific criteria during the dental examination. They are as follows: at least eight stabile teeth preserved in each jaw, a healthy periodontium, and the ability to set a construction bite with the mandibular position at 50–75% of the maximum protrusion and leaving a space between the incisors of about 3–5 mm, enabling free breathing through the mouth [28]. The effectiveness of the device increases with the degree of the mandibular protrusion, but the patient’s tolerance comparably suffers [29]. Studies on the correlation of the therapeutic effect with the degree of protrusion revealed that the latter is the fundamental factor influencing patients’ betterment [30,31]. Attaining 70% of the maximal mandibular protrusion is, according to some authors, a compromise between the device’s effectiveness and the occurrence of potential side effects of its application [32]. In the present study, the mandible was protruded at 60% of its maximal range so that the device was better tolerated and the adverse effects of the MAD application were avoided. Possible adverse effects include painful sensations in the TMJ, changes in overjet and overbite, disorders within Angle’s classes, and changes in the inclination of the maxillary incisor to the cranial base (1/NS) and the angle between the Sella-Nasion-Supramentale (SNB) [33,34]. Regarding the vertical dimension of the construction bite, it is claimed that it should be retained at a minimum level since increasing the vertical dimension through mandibular abduction leads to a down-backward displacement of the tongue, which reduces the patency of the airways [35]. The devices used in the present study facilitated mandibular abduction and prevented mandibular retrusion, but the degree of protrusion may have been insufficient.

The patients’ response to the treatment varied considerably, which could be due to many factors. One of them is the degree of the apnea intensity prior to treatment, which cannot be verified after one PSG examination. According to Aarab et al., apnea intensity varies depending on a particular night—from a normal condition to severe apnea [36]. Response to treatment with a specific method can be conditioned by a varied morphological structure of the craniofacial region and the upper airways, as well as by the duration of the condition and its treatment. 

Undoubtedly, the strength of the study presented here was in the use of polysomnography to assess for OSA and SB. However, the limitations of the study were the small number of patients, the reduction in the number of PSG examinations to one with an MAD and one without an MAD, and the inability to adjust the degree of the mandibular protrusion in the device itself on consecutive nights. 

In this regard, it seems reasonable to conduct similar studies on larger samples with diagnosed OSA and SB and include several PSG examinations with and without MADs to assess changes in the parameters used to describe OSA and SB. Conducting several PSG examinations with an MAD with different degrees of mandibular protrusion is also worth considering.

## 5. Conclusions

The application of MADs in patients with OSA has a beneficial effect on the manifestations of OSA and SB, even though only the number of phasic episodes of bruxism was statistically significant. 

## Figures and Tables

**Table 1:** Inclusion and exclusion criteria.

Inclusion	Exclusion
Age > 18 AHI > 15—moderate-to-severe form of OSA At least eight teeth each in the upper and lower dental arch No periodontal conditions No temporomandibular dysfunctions	Medical: ✓ Sleep/Respiratory disorder other than OSA ✓ Taking of medications disrupting sleep or respiration ✓ Previous treatment with MAD ✓ Morphological disorders of the upper respiratory tract ✓ Mental disorders Dental: ✓ Temporomandibular dysfunctions ✓ Untreated periodontal or mucosal diseases ✓ Absence of at least eight teeth each in the upper and lower dental arch

AHI apnea-hypopnea index, OSA obstructive sleep apnea, MAD mandibular advancement devices.

**Table 2:** Comparison of all of the variables examined before and after the MAD application (N = 8).

Parameter		Mean ± SD	Min–Max
	Before	44.9 ± 18.6	17.5–70.0
AHI	with MAD	33.8 ± 18.5	15.5–66.7
	Before	40.7 ± 17.8	15.9–66.8
ODI	with MAD	25.4 ± 13.9	5.1–50.9
	Before	288.3 ± 122.2	108.0–463.0
Apneas + Hypopneas	with MAD	197.5 ± 92.0	78.0–378.0
	Before	166.8 ± 66.0	68.0–271.0
Obstructive apneas	with MAD	93.9 ± 61.9	22.0–199.0
	Before	24.1 ± 49.9	0.0–144.0
Mixed apneas	with MAD	1.9 ± 2.6	0.0–6.0
	Before	94.4 ± 101.2	36–342
Hypopneas	with MAD	94.1 ± 72.4	34–258
	Before	193.9 ± 97.7	69.0–378
Apneas	with MAD	103.4 ± 59.2	22.0–199.0
	Before	93.3 ± 1.6	90.5–95.5
Mean SpO_2_	with MAD	93.8 ± 0.9	92.5–95.4
	before	78.4 ± 4.5	69.0–83.0
Min SpO_2_	with MAD	80.4 ± 4.5	74.0–86.0
	before	4.4 ± 6.3	2.0–18.4
BEI	with MAD	3.3 ± 3.4	0.0–9.8
	before	36.0 ± 40.3	1.0–114.0
Episodes of bruxism	with MAD	11.8 ± 11.2	0.0–34.0
	before	8.4 ± 11.9	0.0–35.0
Phasic	with MAD	2.0 ± 3.4	0.0–10.0
	before	17.6 ± 20.5	1.0–64.0
Tonic	with MAD	9.0 ± 11.9	0.0–33.0
	before	10.0 ± 15.0	0.0–39.0
Mixed	with MAD	0.8 ± 0.7	0.0–2.0

AHI apnea-hypopnea index, ODI oxygen desaturation index, BEI bruxism episodes index, MAD mandibular advancement devices.

**Table 3:** Mean changes of the values of the parameters assessed with polysomnographic examinations following MAD applications.

Parameter	MAD	*p*/T-Student Test	*p*/Wilcoxon Test
AHI	−11.05 ± 8.3	0.007	0.012
ODI	−15.28 ± 12.0	0.009	0.017
Apneas + Hypopneas	−90.75 ± 59.07	0.003	0.012
Obstructive apneas	−72.86 ± 68.94	0.020	0.036
Mixed apneas	−22.25 ± 50.41	-	0.237
Hypopneas	−0.25 ± 47.06	-	0.779
Apneas	−90.5 ± 74.09	0.011	0.012
Mean SpO_2_	0.44 ± 0.95	0.230	0.441
Min SpO_2_	2.0 ± 3.55	0.155	0.260
BEI	−1.14 ± 7.9	-	0.779
Episodes of bruxism	−24.25 ± 42.41	0.150	0.233
Phasic	−6.38 ± 8.75	-	0.034
Tonic	−8.63 ± 23.87	-	0.401
Mixed	−9.25 ± 15.04	-	0.068

AHI apnea-hypopnea index, ODI oxygen desaturation index, BEI bruxism episodes index.

**Table 4:** Correlations of the dependent samples with and without an MAD (N = 8).

Parameter	Correlation	Significance
AHI	0.898	0.002
ODI	0.740	0.036
apneas + hypopneas	0.885	0.003
obstructive apneas	0.420	0.300
mixed apneas	−0.165	0.697
WE confirmhypopneas	0.905	0.002
apneas	0.653	0.079
mean SpO_2_	0.846	0.008
min SpO_2_	0.692	0.057
BEI	−0.266	0.524
episodes of bruxism	−0.054	0.899
phasic episodes of bruxism	0.948	0.000
tonic episodes of bruxism	−0.016	0.970
mixed episodes of bruxism	−0.081	0.849

AHI apnea-hypopnea index, ODI oxygen desaturation index, BEI bruxism episodes index.

## Data Availability

The data presented in the study are available on reasonable request from the authors of this article.
